# Effects of dry mouth lozenges on the surface morphology and color stability of restorative materials

**DOI:** 10.1007/s10266-026-01315-5

**Published:** 2026-01-14

**Authors:** Aslı Aşık, Sibel Acar, Dilşah Çoğulu

**Affiliations:** 1 Department of Pediatric Dentistry, Faculty of Dentistry, İzmir Tınaztepe University, İzmir, Turkey; 2 Department of Pediatric Dentistry, Faculty of Dentistry, Ege University, İzmir, Turkey

**Keywords:** Dry mouth, Saliva substitute, Color stability, Acidity, Surface morphology, Spectrophotometry

## Abstract

**Objectives:**

Lozenges are frequently recommended to stimulate saliva for patients suffering from dry mouth to improve the quality of their lives. This study aimed to investigate the effects of dry mouth lozenges with varying acidity levels on the color stability and surface morphology of resin-based restorative materials.

**Materials and methods:**

Eighty specimens, comprising a composite (*n* = 40) and a compomer (*n* = 40), were prepared using a standardized mold. The baseline color (T0) of each specimen was measured using the CIE (L*,a*,b*) color assessment system with Vita Easy Shade Compact. The specimens were then divided into eight groups: Group-1: Composite-ACT, Group-2: Composite-TheraBreath, Group-3: Composite-Biotene, Group-4: Composite-Xylimelts, Group-5: Compomer-ACT, Group-6: Compomer-TheraBreath, Group-7: Compomer-Biotene, and Group-8: Compomer-Xylimelts. Each lozenge was dissolved in 10 mL of distilled water, and the pH of the solutions was measured using a pH meter. Over a period of one week, the specimens were immersed in the respective lozenge solutions within Eppendorf tubes, and color measurements were repeated on the seventh day (T1). Subsequently, the color changes (ΔE*) over time were statistically analyzed. Randomly selected specimens from each group were examined using scanning electron microscopy (SEM) after 7 days to evaluate surface morphology. For the statistical analysis, a significance level of *p* < 0.05 was applied.

**Results:**

The pH levels of the dry mouth lozenges tested were as follows: ACT (8.53), TheraBreath (3.55), Biotene (5.34), and Xylimelts (8.14). After 7 days, the color change (ΔE) in the groups was recorded as: G1 (4.06 ± 2.29), G2 (2.63 ± 1.67), G3 (5.97 ± 4.32), G4 (4.34 ± 3.00), G5 (12.76 ± 2.73), G6 (15.53 ± 5.06), G7 (11.09 ± 4.70), and G8 (4.85 ± 1.77). The two-way ANOVA revealed a significant interaction between restorative material and lozenge type (*p* < 0.001). Compomer materials exhibited significantly greater color change than composite materials when exposed to ACT, TheraBreath, and Biotene lozenges (*p* < 0.05). However, no significant difference was observed between materials with Xylimelts lozenge (*p* > 0.05). Among compomers, Xylimelts caused significantly less discoloration compared to other lozenges, while composite materials showed no significant color changes across different lozenge types.

**Conclusion:**

The choice of restorative material is critical for pediatric patients who use dry mouth lozenges. This study demonstrates that while composite resins maintained stable color regardless of the lozenge type, compomers were highly susceptible to discoloration when exposed to certain lozenges. Therefore, composite resin is a more reliable choice for esthetic restorations in patients using these specific lozenge products.

## Introduction

Saliva performs a range of essential functions, including facilitating remineralization, inhibiting demineralization, providing lubrication for oral tissues, aiding in the digestive process, enhancing gustatory perception, assisting in the formation of the food bolus, and exhibiting antimicrobial properties, including antiviral, antifungal, and antibacterial activities. Its composition is predominantly water but also includes proteins, glycoproteins, mineral ions, and enzymes. In addition, saliva contains gingival crevicular fluid, non-adherent microorganisms, food debris, and various human cells, such as leukocytes and epithelial cells. Under normal physiological conditions, the average daily production of whole saliva ranges from 1 to 1.5 L [1]. Approximately 90% of the total saliva volume is secreted by the three major paired salivary glands: the parotid, submandibular, and sublingual glands. The normal unstimulated salivary flow rate ranges between 0.3 and 0.5 mL/min, whereas the stimulated flow rate increases to approximately 1.5 to 2.0 mL/min under conditions such as mastication or gustatory stimulation [2].

Xerostomia, commonly referred to as dry mouth, denotes the subjective sensation of oral dryness. While not always directly associated with salivary gland dysfunction, xerostomia is frequently linked to a marked reduction in unstimulated salivary secretion. Salivary gland hypofunction is clinically defined by an unstimulated salivary flow rate of less than 0.1 mL/min or a stimulated flow rate below 0.5 mL/min. Additionally, a 50% reduction in an individual’s baseline salivary flow rate is also classified as hypofunction, reflecting the broad variability within normal salivary production ranges [3, 4]. A significant reduction in salivary production can lead to considerable discomfort, impairments in mastication and swallowing, and an elevated risk of developing oral pathologies, such as dental caries. The protective functions of saliva, including its roles in buffering acids, facilitating remineralization, and maintaining oral hygiene through antimicrobial activity, are diminished, thereby increasing disease susceptibility [5].

While dry mouth is a well-documented condition in adults, it has garnered relatively less attention in pediatric populations. A common misconception within the dental field is that children, who frequently exhibit excessive salivation, do not experience salivary hypofunction. However, a healthy child typically demonstrates a stimulated salivary flow rate exceeding 1.0 mL/min, which is moderately higher than the rate observed in adults (approximately 0.5 mL/min). Despite this, pediatric patients can experience xerostomia due to various etiologies, including dehydration from inadequate fluid intake, excessive consumption of caffeinated beverages (e.g., cola), underlying renal conditions necessitating hemodialysis, and eating disorders [6].

The induction of xerostomia by various pharmacological agents is a widely acknowledged and well-characterized occurrence in pediatric patients. Antihistamines, frequently prescribed for allergic rhinitis [7], anti-anxiety medications used in the treatment of obsessive–compulsive disorder [8], amphetamines for managing attention-deficit/hyperactivity disorder [9], and chemotherapeutic agents administered to oncology patients [10–12] are all associated with reduced salivary flow.

Chronic xerostomia significantly increases the risk of developing dental caries, demineralization, tooth hypersensitivity, enamel erosion, fungal infections, and various other oral health complications, all of which can adversely affect an individual’s overall quality of life [3]. In pediatric patients with xerostomia, management primarily focuses on symptomatic treatment, with each intervention aimed at improving quality of life and preventing oral health complications. For children experiencing chronic salivary gland dysfunction, long-term management is essential and typically includes the use of salivary substitutes, casein phosphopeptide-amorphous calcium phosphate (CPP-ACP), fluoride varnish applications, periodic chlorhexidine treatments, strict dietary control, and regular dental visits to prevent premature loss of both primary and permanent teeth. Patients are encouraged to take small, frequent sips of water and maintain oral moisture. Parasympathomimetic agents, available as gum, lozenges, candies, rinses, sprays, and gels, are commonly used to alleviate symptoms of dry mouth. For pediatric patients undergoing oncological treatments, additional recommendations include the use of sugar-free gum or lozenges, sucking tablets, specialized toothpaste for dry mouth, saliva substitutes, regular hydration, alcohol-free oral rinses, and oral moisturizers to mitigate xerostomia symptoms [13, 14].

Saliva substitutes necessitate consistent and regular application, with over-the-counter formulations available in various forms, each differing in composition, pH levels, and viscosities. Dry mouth lozenges may be utilized throughout the day as needed, following the manufacturer’s guidelines. These lozenges are designed to be held in the oral cavity until fully dissolved by saliva. Common ingredients in dry mouth lozenges include isomalt, xylitol, and citric acid, which are selected for their capacity to stimulate salivary flow and maintain oral moisture [15].

The current body of literature includes a limited number of studies assessing the impact of various saliva substitute formulations on hard dental tissues. Notably, research has focused on evaluating the potential erosive effects of these products, particularly due to their acidic properties, on enamel and dentin. However, there is a notable deficiency in research examining the effects of saliva substitutes on the physical and esthetic properties of resin-based restorative materials. Furthermore, the long-term performance of restorative materials is fundamentally governed by their interaction with the oral chemical environment. Contemporary resin-based materials, especially polyacid-modified composites (components) and bioactive formulations, are not inert; their composition is designed for specific interactions, which may include water sorption, ion exchange, or surface reactivity. As noted in a recent comprehensive review [16], this inherent chemical activity means that their physical and esthetic stability—such as color fidelity and surface smoothness—can be significantly influenced by prolonged or repeated exposure to non-physiological chemical challenges. The oral cavity of patients using dry mouth lozenges represents a prime example of such a controlled, yet clinically relevant, chemically active milieu. Addressing this gap, the present study aims to evaluate the impact of various dry mouth lozenges on the surface roughness and color stability of composite and compomer restorations. The research question guiding this study is whether the use of dry mouth lozenges induces surface roughening and discoloration in compomer and composite restorative materials. Furthermore, if such effects are observed, the study aims to determine whether the specific composition of the lozenges contributes to these alterations in surface texture and color stability. The null hypothesis posits that dry mouth lozenges exert no significant effect on the surface roughness or color stability of composite and compomer restorative materials.

## Material and methods

The four dry mouth lozenges evaluated in this study are listed in Table 1, while the resin-based restorative materials assessed for changes in color and surface texture are outlined in Table 2. A power analysis was performed using the G*Power 3.1.9.4 software (Heinrich Heine University, Düsseldorf, Germany) to determine the appropriate sample size for the study. Based on the analysis, a total sample size of *n* = 80 was calculated (*α* = 0.05, 1–β = 0.80, effect size = 0.4).

**Table 1:** Dry mouth lozenges used in the present study

Pediatric supplements	Manufacturer	Ingredients	Lot No
ACT dry mouth lozenges	Chattem, Inc., Sanofi Company,2219, Chattanooga, TN,USA	Isomalt, xylitol, glycerin, water, natural and artificial flavors, sucralose, FD&C blue 1	100038183
Biotene dry mouth lozenges	GSK Consumer Healthcare, Warren, NJ 07059,USA	Isomalt, xylitol, water, glycerin, sodium citrate, natural and artificial flavor, pectin, sucralose, FD&C blue 1	100035563
TheraBreath dry mouth lozenges	Church&Dwight Co.,Inc.,Ewing, NJ,08628, USA	Sorbitol, maltodextrin, flavors, magnesium stearate, xylitol, malic acid, spilanthes acmella flower extract blend, tartaric acid, citric acid, zinc gluconate, stevia rebaudiana leaf extract	TL319543
OraCoat Xylimelts dry mouth lozenges	Quest Products,LLC,8201, Pleasant Prairie, WI 53158, USA	Xylitol, acacia gum, cellulose gum, magnesium stearate, sodium bicarbonate, calcium carbonate	551062026

**Table 2:** Pediatric resin-based restorative materials used in the present study

Restorative materials	Type	Composition	(%)Volume	Lot number	Manufacturer
Filtek^™^ Z550	Composite resin	Bis-GMA, Bis-EMA, PEGDMA, TEGDMA,UDMA, surface-modified zirconia/silica fillers 3000 nm (3 μm or less), nonagglomerated/non-aggregated surface-modified silica particles 20 nm	68 vol%	10297071	3 M/ESPE, St.Paul, MN, USA
Glasiosite^®^	Compomer	Bis-GMA, diurethane-dimethacrylate, TEGDMA, BHT	78 vol%	2317263	Voco, Cuxhaven, Germany

A total of forty specimens of composite resin (Filtek^™^ Z550, 3 M ESPE, St. Paul, USA) and forty specimens of compomer (Glasiosite^®^, Voco, Cuxhaven, Germany) were fabricated using standardized molds with dimensions of 4 mm in diameter and 2 mm in height. The specimens were prepared by considering factors that would facilitate coloration. All specimen preparation, polishing, and immersion procedures were carried out by the same operator under standardized conditions using uniform molds, a fixed polishing protocol, and equal solution volumes to ensure reproducibility. Each specimen was prepared in a single layer, leveled, and polymerized for 20 s using a LED curing light (T-LED, MyRay, Italy) set to a standard mode with a 1250-mW/cm^2^ light intensity. The output intensity of the LED curing unit was verified with a digital radiometer (Bluephase Meter II, Ivoclar Vivadent, Liechtenstein) prior to specimen preparation to ensure consistent polymerization conditions. To create a smooth surface, transparent tape was used during specimen preparation, which prevented the formation of the undesirable oxygen inhibition layer and ensured polishing of the specimens. After specimen preparation, the specimens were immersed in distilled water for 24 h, allowing for the removal of any residual monomer [17] and the completion of the polymerization process [18]. Following polymerization, the specimens were polished using aluminum oxide disks (Sof-Lex, 3 M ESPE, St. Paul, MN, USA). Initial color measurements were then taken to establish baseline values (T0). A total of 8 groups took part in the present study and they are as follows: Group 1-Composite-ACT, Group 2-Composite-TheraBreath, Group 3-Composite-Biotene, Group 4-Composite-Xylimelts, Group 5-Compomer-ACT, Group 6-Compomer-TheraBreath, Group 7-Compomer-Biotene and Group 8-Compomer-Xylimelts.

**Fig. 1:**
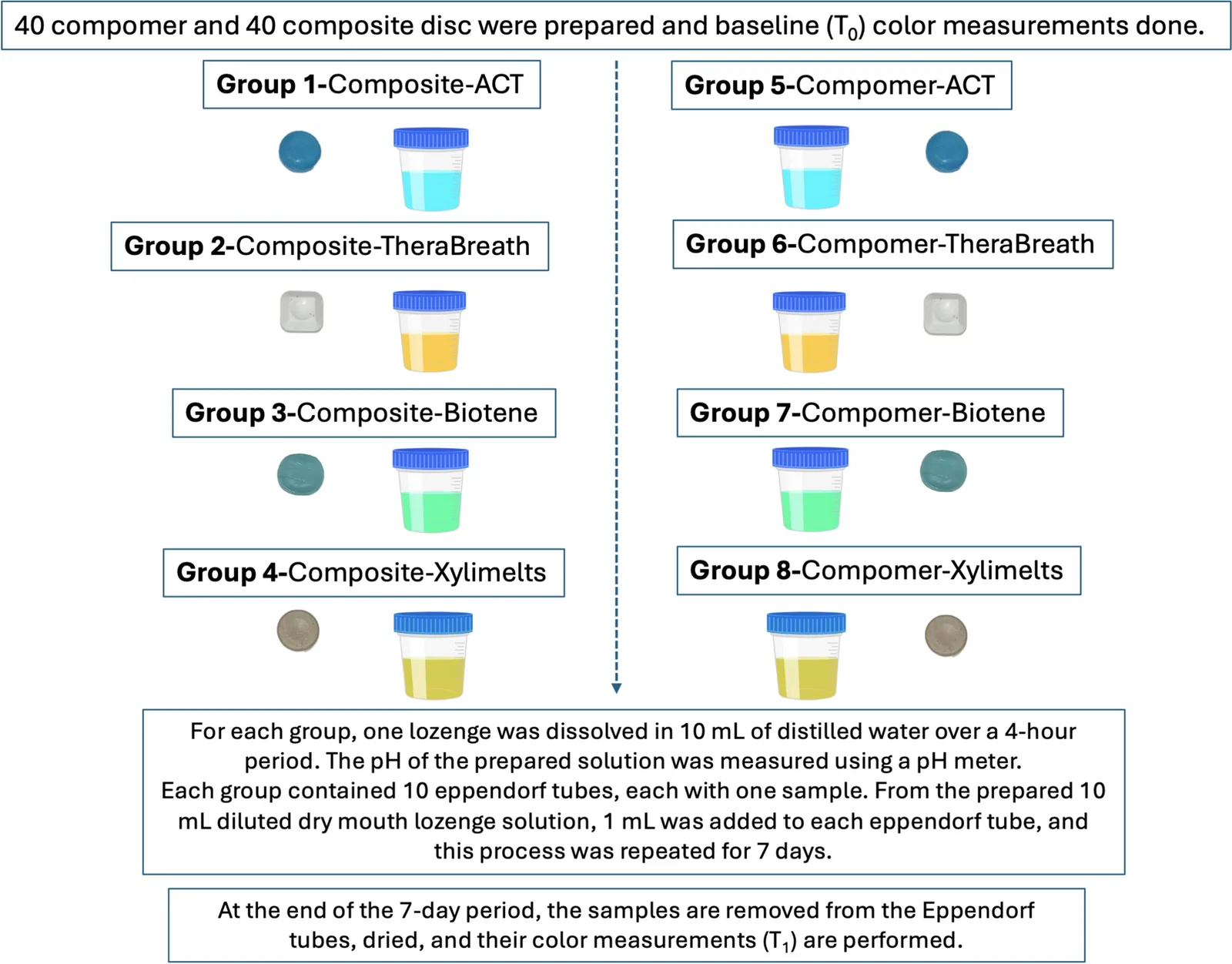
Experimental process of the study

The specimens were placed in individual Eppendorf tubes. Each experimental group consisted of 10 tubes, with one specimen per tube, and the tubes were labeled according to their corresponding groups. The ‘top’ surface, obtained against a transparent matrix strip during polymerization, was designated as the exposed surface for immersion and subsequent color measurements. To ensure consistent identification and orientation during repeated evaluations, a small notch was created on the bottom surface of each specimen using a high-speed aerator. For each dry mouth lozenge group (G1/G5: ACT, G2/G6: TheraBreath, G3/G7: Biotene, G4/G8: Xylimelts), a single lozenge (2 g) was dissolved in 10 ml of distilled water for a duration of 4 h. Subsequently, 1 ml of the prepared lozenge solution was added to each Eppendorf tube. This procedure was repeated daily for a total of 7 days. Specimens were immersed in the lozenge solutions continuously for 24 h per day over 7 days, and the solutions were refreshed daily to maintain chemical stability and prevent contamination. On the 7th day (T1), the specimens were removed from the solution, rinsed under running distilled water**,** dried, and subjected to colorimetric analysis. The experimental workflow is visually summarized in Fig. 1.

The use of spectrophotometric ΔE analysis to quantify color changes in restorative materials following exposure to chemical solutions is a well-established method for evaluating aesthetic stability, as demonstrated in studies assessing modified composite systems under various oral conditions [19–21].

Before performing measurements, the Vita Easyshade spectrophotometer was calibrated using the manufacturer-provided calibration tool, as recommended by the manufacturer. Color assessments were conducted under standardized D65 illumination conditions, against a black background. Color values were measured using the CIE L*a*b* system, with each specimen measured three times and the mean value recorded. Color change (ΔE*) between different time points was then calculated using the following formula:

$$\Delta {\mathrm{E}}^{ * } = \,\left[ {\left( {\Delta {\mathrm{L}}^{ * } } \right)^{2} + \left( {\Delta {\mathrm{a}}^{ * } } \right)^{2} + \left( {\Delta {\mathrm{b}}^{ * } } \right)^{2} } \right]^{{{\raise0.7ex\hbox{$1$} \!\mathord{\left/ {\vphantom {1 2}}\right.\kern-0pt} \!\lower0.7ex\hbox{$2$}}}}$$

where ΔL*, Δa*, and Δb* represent the differences in L*, a*, and b* values, respectively. According to Paravina et al., a ΔE* value of 2.7 units represents the clinical acceptability threshold at a 50:50% level, as expected to become the ISO standard for dental material color evaluation [20].

Scanning electron microscopy (SEM) is a standard technique for the qualitative assessment of surface morphology changes in dental materials following chemical or aging challenges, allowing for direct visualization of degradation, erosion, or topographic alterations [21]. To obtain a qualitative visual illustration of potential surface alterations, a single representative specimen from each experimental group was randomly selected for surface analysis via SEM. SEM images (Apreo S, Thermo Fisher Scientific, USA) were obtained after the 7-day immersion period. For SEM analysis, specimens were gold sputtered after completion of the color measurements to improve conductivity, and images were taken at × 500, × 1000, × 2000, × 5000, and × 10,000 magnifications.

### Statistical analysis

Data analysis was performed using IBM SPSS Statistics 25.0 (Armonk, NY, USA). Descriptive statistics, including mean, standard deviation, median, minimum, and maximum, were calculated for the color change values (ΔE*). The normality of data distribution was verified with the Shapiro–Wilk test, and the homogeneity of variances was assessed using Levene’s test. The primary analysis to evaluate the effects of restorative material and lozenge type on color change (ΔE*) was conducted using two-way analysis of variance (two-way ANOVA). This statistical model was selected to assess both the main effects of each independent variable and their interaction effect. The two-way ANOVA model had 72 degrees of freedom for the error term (df_error = 72), calculated based on the total sample size (*N* = 80) and the number of groups (*k* = 8). When a significant interaction was identified, simple main effects analysis was performed to examine the effect of one factor at each level of the other factor. Post-hoc comparisons for significant main effects were conducted using Tukey’s HSD test. The significance level was set at *p* < 0.05 for all analyses.

## Results

Each lozenge was dissolved in 10 mL of distilled water over a period of 4 h, after which the pH of the resulting solutions was measured using a calibrated pH meter (HI 5521, Hanna Instruments, USA) to ensure accurate pH readings. The measured pH values for the respective lozenge solutions were as follows: the ACT solution demonstrated a pH of 8.53, the TheraBreath solution presented a pH of 3.55, the Biotene solution recorded a pH of 5.34, and the Xylimelts solution exhibited a pH of 8.14. Descriptive statistics for the color change (ΔE*) values are presented in Table 3.

**Table 3:** Descriptive statistics for color change (ΔE*) values

Restorative material	Lozenges	Min.-max	Mean ± SD	Median
Composite	ACT	1.62–9.5	4.06 ± 2.29	3.94
	TheraBreath	0.53–5.37	2.63 ± 1.67	2.47
	Biotene	1.12–15.19	5.97 ± 4.32	3.96
	Xylimelts	1.99–12.17	4.34 ± 3.00	3.32
Compomer	ACT	9.0–17.35	12.76 ± 2.73	13.17
	TheraBreath	10.79–27.97	15.53 ± 5.06	14.29
	Biotene	2.64–16.8	11.09 ± 4.70	11.38
	Xylimelts	2.64–8.35	4.85 ± 1.77	4.31

Given that pH is a fixed property of each lozenge solution, a traditional correlation analysis with individual specimen data is not statistically appropriate. Instead, to explore trends at the group level, the mean ΔE values for each material–lozenge combination were considered in relation to their corresponding solution pH (presented in Table 3). Visual inspection of this data does not reveal a consistent pattern where higher or lower pH directly predicts the magnitude of color change. For example, for compomer, the two lozenges with similar basic pH (ACT: 8.53 and Xylimelts: 8.14) resulted in markedly different mean ΔE* values (12.76 vs. 4.85). Conversely, the most acidic lozenge (TheraBreath, pH = 3.55) did not produce the highest mean ΔE* for composite.

Two-way ANOVA revealed a statistically significant main effect of restorative material (F (1, 72) = 154.73, *p* < 0.001), a significant main effect of lozenge type (F (3, 72) = 13.42, *p* < 0.001), and a statistically significant interaction between material and lozenge (F(3, 72) = 17.62, *p* < 0.001). The significant interaction indicates that the effect of lozenge type on color change depended on the restorative material. Simple main effects analyses with Bonferroni corrections were performed to interpret this interaction.

### Effect of material within each lozenge

For ACT, TheraBreath, and Biotene lozenges, compomer showed significantly higher ΔE* values than composite (*p* < 0.001 for all). However, for Xylimelts lozenge, there was no statistically significant difference in color change between composite and compomer (*p* = 0.190).

### Effect of lozenge within each material

**Fig. 2:**
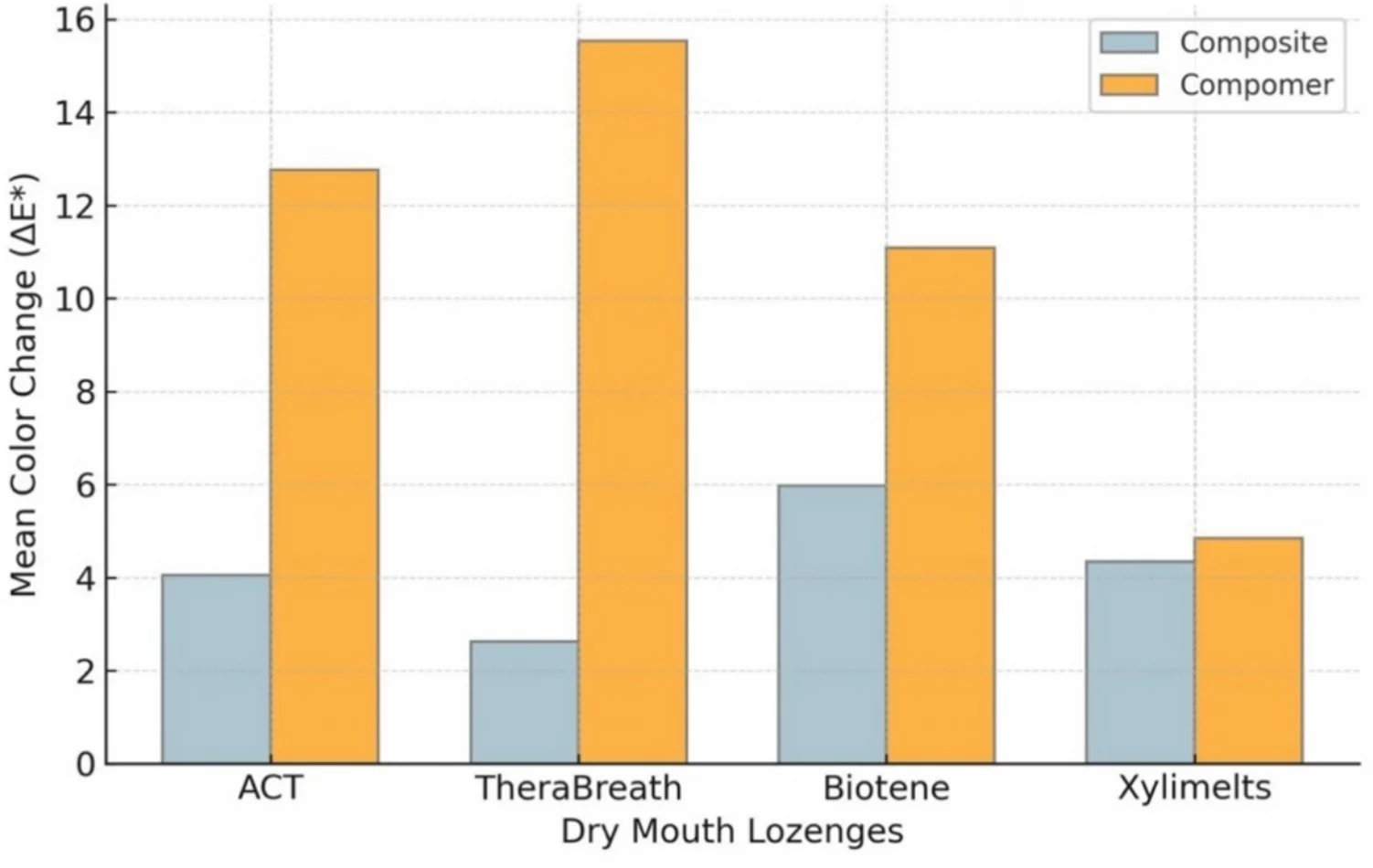
Mean color change (ΔE*) of composite and compomer materials after exposure to different dry mouth lozenges

For composite material, there was no statistically significant difference in color change among the different lozenges (*p* = 0.111). For compomer material, there was a statistically significant difference in color change based on the lozenge (*p* < 0.001). Post-hoc Tukey tests revealed that Xylimelts lozenge resulted in significantly lower ΔE* values compared to ACT (*p* = 0.003), TheraBreath (*p* < 0.001), and Biotene (*p* = 0.035) lozenges. A graphical representation of the interaction between restorative material and lozenge type on color change is provided in Fig. 2.

To assess the clinical relevance of the measured color changes, the data were interpreted using the perceptibility and acceptability threshold of ΔE = 2.7, as proposed by Paravina et al. [18]. The mean ΔE* values for all compomer groups exposed to ACT (12.76), TheraBreath (15.53), and Biotene (11.09) lozenges substantially exceeded this acceptability threshold. In contrast, the mean ΔE* for compomer exposed to Xylimelts (4.85) was closer to, yet still above, the threshold. For composite materials, the mean ΔE* values across all lozenge groups (ACT: 4.06, TheraBreath: 2.63, Biotene: 5.97, Xylimelts: 4.34) were more variable; notably, the value for TheraBreath was below the 2.7 threshold, while the others exceeded it.

**Fig. 3:**
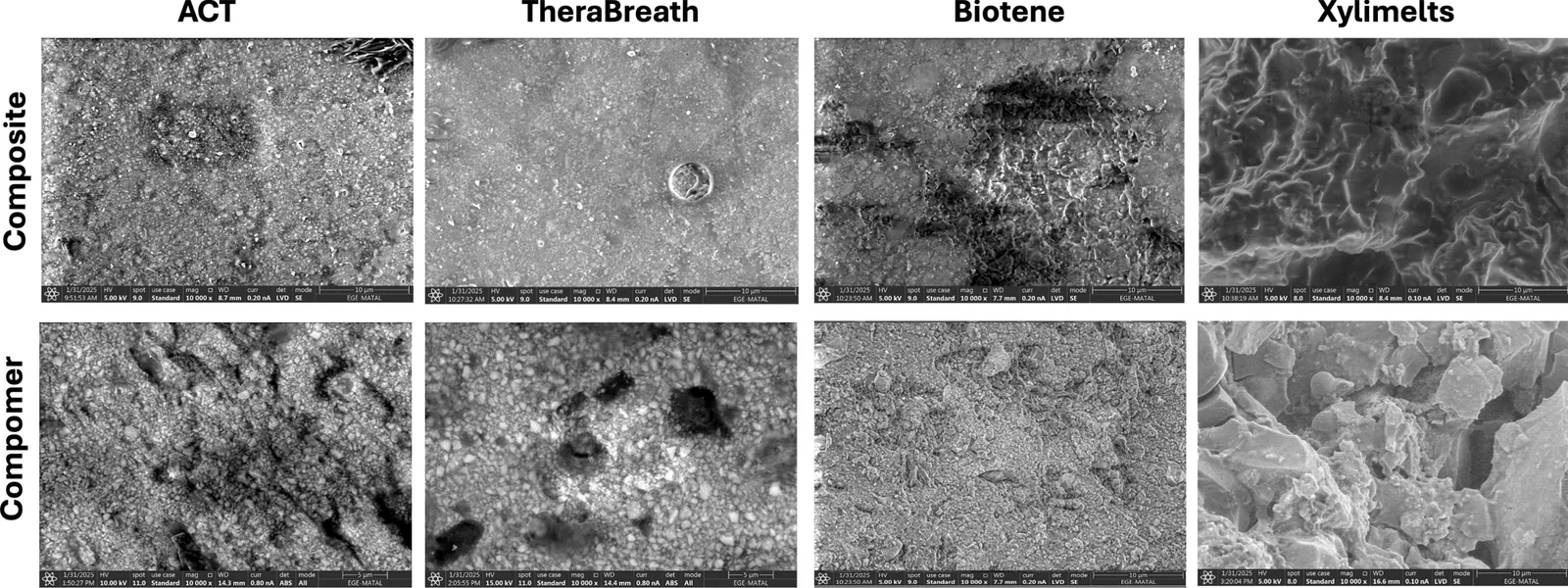
SEM examination of restorative materials following the administration of dry mouth lozenges

The SEM images obtained at 10,000 × magnification are presented in Fig. 3. Qualitative SEM analysis suggested that dry mouth lozenges led to surface alterations in both composite and compomer materials, irrespective of the group. Notably, the surfaces of compomers exhibited more pronounced changes as a consequence of the use of dry mouth lozenges.

## Discussion

This study investigated the effects of various dry mouth lozenges on the color stability and surface morphology of a composite and a compomer restorative material. The key finding was a significant interaction between material type and lozenge composition, indicating that the esthetic outcome is not determined by the lozenge’s pH alone. While the composite resin demonstrated superior and more consistent color stability across all tested lozenges, the compomer exhibited pronounced, clinically unacceptable discoloration (ΔE > 2.7) when exposed to ACT, TheraBreath, and Biotene formulations. These results align with the limited prior research focusing on the erosive potential of saliva substitutes on natural teeth but provide novel data on their impact on common esthetic restorations [15, 22–25]. Furthermore, the susceptibility of the compomer contrasts with the stability of the composite, a distinction that can be understood through the fundamental differences in their polymer matrix chemistry and water sorption behavior, as discussed below.

Color stability is defined as the ability of restorative materials to maintain their initial color under various factors in a specific environment and duration [19]. Discolorations can be classified as either intrinsic or extrinsic. Intrinsic discolorations are influenced by the resin material's composition, water absorption, degree of polymerization, filler, and particle characteristics. Extrinsic discolorations, on the other hand, result from the absorption of staining agents [26, 27]. Resin materials with lower filler content exhibit poorer color stability and are more susceptible to discoloration. This is due to the increased water absorption at the matrix–filler interface caused by the reduced filler content. The separation of the filler from the resin and the hydrolytic degradation of the filler lead to an increased propensity for extrinsic discoloration [28]. The interface between the resin and filler particles in composite materials is a critical weak point, exhibiting high sensitivity to water absorption. Consequently, hydrolytic deterioration of this interface may alter the scattering behavior of light by the particles [29]. The standardized preparation protocol, including measures taken to minimize the oxygen inhibition layer, may also have influenced the color stability results observed in this study.

The composite material (Filtek™ Z550, 3 M ESPE, USA) used in the study contains bisphenol A diglycidyl methacrylate (Bis-GMA), bisphenol A diglycidyl methacrylate ethoxylated (Bis-EMA), polyethylene glycol dimethacrylate (PEGDMA), triethylene glycol dimethacrylate (TEGDMA), and urethane dimethacrylate (UDMA). Bis-EMA is more resistant to discoloration due to its hydrophobic structure. [30, 31] Similarly, UDMA causes less water absorption compared to Bis-GMA and is more resistant to staining. UDMA contributes to color stability due to its hydrophobic nature and lower water sorption compared to Bis-GMA, thereby reducing hydrolytic degradation and extrinsic staining. Furthermore, the absence of aromatic structures in UDMA lowers the risk of chromophore formation, which further explains its superior resistance to discoloration. The compomer material (Glasiosite^®^, Voco, Cuxhaven, Germany) used in our study contains Bis-GMA but lacks more hydrophobic monomers like Bis-EMA and UDMA, which are present in the composite. This compositional difference, combined with the polyacid-modified resin matrix of compomers, likely increases their water sorption and susceptibility to surface degradation. This explains the significantly greater color change observed in compomers compared to composites when exposed to ACT, TheraBreath, and Biotene lozenges.

The observed vulnerability of the compomer material can be further contextualized by comparing it to the behavior of other chemically active restorative systems, such as conventional and resin-modified glass ionomer cements (RMGICs). Similar to compomers, these materials possess polyacid-based matrices designed for ion release and fluoride recharge. Studies on RMGICs and related bioactive hybrids exposed to acidic challenges have documented analogous susceptibility to surface degradation and alterations in physical properties. For instance, research on phytomedicine-modified glass ionomers highlights that while bioactive modifications can enhance certain functions, the underlying ionic matrix remains sensitive to low-pH environments, which can affect surface integrity and potentially influence long-term color stability [32]. This parallel underscores a common theme: the very chemical reactivity that confers bioactivity to materials like glass ionomers and compomers (e.g., fluoride release, ion exchange) may also render them more susceptible to degradation in chemically challenging, non-physiological milieus, such as those created by certain acidic saliva substitutes. In contrast, the more hydrophobic and stable polymer network of conventional composites, as demonstrated in the present study, appears to provide a more robust barrier against such chemical challenges, prioritizing structural and esthetic stability over ionic functionality [33].

The dry mouth lozenges utilized exhibit diverse compositional characteristics and varying degrees of acidity. While ACT (pH = 8.53) and Xylimelts (pH = 8.14) possess a basic nature, TheraBreath (pH = 3.55) demonstrates an acidic profile. This is primarily attributed to the presence of malic acid, tartaric acid, and citric acid in the formulation of TheraBreath. In contrast, Xylimelts exhibits a basic property due to the sodium bicarbonate and calcium carbonate in its composition. Furthermore, these constituents with known remineralizing effects are also believed to be effective in color stability. The inclusion of calcium in acidic lozenges has been found to substantially diminish their erosive properties and prevent the demineralization of hydroxyapatite, even in instances where the pH temporarily drops below the critical level for enamel [34]. While Biotene (pH = 5.34) lozenges are similar in composition to ACT lozenges, they exhibit a more acidic characteristic due to the presence of sodium citrate, which is formed from the combination of citric acid and sodium. Acidic environments can lead to the solubilization of restorative materials. This increased solubility causes surface erosion and dissolution, thereby altering the surface roughness and hardness properties of the materials. Decreased oral pH leads to increased absorption of resin-based materials, and surface degradation of these materials subsequently results in extrinsic discoloration. When the surface is smooth and has fewer surface defects, the restorative material demonstrates better color resistance [35, 36].

A key finding of this study is that the discoloration potential of a dry mouth lozenge cannot be predicted by its pH alone. It is critical to acknowledge a methodological consideration: as pH is a fixed value for each lozenge formulation, conducting a formal statistical correlation with individual specimen color change values is not valid. Our exploratory, group-level comparison indicates that factors beyond simple acidity or alkalinity are decisive. For instance, the similar basic pH of ACT and Xylimelts led to vastly different effects on compomer discoloration. This underscores that the specific chemical composition of the lozenge (e.g., the presence of specific acids, buffering agents like sodium bicarbonate, or remineralizing ions like calcium) and its interaction with the restorative material’s polymer matrix and fillers are the primary drivers of color change, rather than the solution’s pH per se. Interestingly, qualitative SEM analysis suggested that the most acidic lozenge (TheraBreath, pH = 3.55) produced the most distinct surface alterations, such as hole-like structures, particularly on the compomer. This disconnect indicates that while low pH may contribute to surface erosion and roughness—a potential precursor for long-term staining—the immediate color change measured in this study was more strongly influenced by the specific material-lozenge interaction.

The significant interaction effect between material and lozenge type, combined with the clinical threshold analysis, underscores that the clinical performance of a restorative material cannot be assessed in isolation. The compomer’s high susceptibility to discoloration from some lozenges (ACT, TheraBreath, Biotene) resulted in mean ΔE* values (11.09 to 15.53) far exceeding the clinical acceptability limit (ΔE* > 2.7), suggesting that such combinations would likely lead to clinically perceptible and unacceptable esthetic compromise over time. Conversely, while some composite groups also exceeded the threshold, the material demonstrated overall greater color stability, with one group (TheraBreath) yielding a mean ΔE* below 2.7. The exceptional performance of both materials with Xylimelts—particularly for compomer, which showed a markedly lower ΔE* (4.85) compared to other lozenges—could be attributed to its basic pH and the presence of remineralizing agents like calcium carbonate, which may have a neutralizing and protective effect on the restoration surface.

The addition of acidic components to dry mouth lozenge formulations, despite causing surface damage to dental hard tissues and restorative materials, is intended to stimulate and increase salivary flow [37]. However, it should not be overlooked when saliva substitutes recommend to patients the advantages and disadvantages of the acidic components of saliva substitutes.

This study provides, to our knowledge, novel qualitative evidence from SEM imaging on surface alterations in restorative materials following exposure to dry mouth lozenges, an area with scant prior visual data. The SEM images obtained in this study provide qualitative illustrative evidence. Notably, the compomer material exhibited more pronounced surface degradation across all groups, which was qualitatively associated with its greater susceptibility to discoloration. The most distinct surface alterations, such as hole-like structures, were observed on compomer specimens exposed to the most acidic lozenge (TheraBreath, pH = 3.55). This visual finding is consistent with its high erosive potential**.**

The limitations of the present in vitro study include the absence of key factors that could influence discoloration in a clinical setting, such as temperature fluctuations, masticatory forces, and individual oral hygiene habits. These variables are inherent to the oral environment where restorative materials are placed. To address these limitations, all specimens in the study were treated and compared under standardized conditions. A primary consideration is the experimental immersion protocol itself. The 7-day, 24-h continuous immersion in freshly prepared lozenge solutions was designed as an accelerated aging model. Its rationale is to condense the cumulative chemical exposure from intermittent, long-term lozenge use (potentially over months or years) into a controlled, observable timeframe. This approach is necessary to detect material interactions within a feasible experimental period and is a common methodology in studies assessing the degradation of dental materials [17, 21]. However, it is crucial to interpret the results within this context. This model does not directly simulate the dynamic clinical conditions of intermittent lozenge use, where salivary clearance, dilution, and oral pH buffering would occur. Therefore, while the study robustly identifies the potential for certain lozenge-material combinations to cause discoloration and surface change, the extent and rate of change observed may be amplified compared to a clinical setting. Another constraint of this study is the relatively short experimental duration of 7 days. However, this limitation was partly addressed by maintaining the specimens under continuous 24-h immersion with daily renewal of lozenge solutions, representing an accelerated aging model. This protocol was intended to mimic the cumulative long-term impact of lozenge use within a condensed timeframe, although further research with extended durations is still warranted. One more limitation of this study is the use of the CIELab (ΔE*) formula instead of the more perceptually accurate CIEDE2000 method. This approach was chosen to maintain comparability with previous studies employing similar lozenge-immersion protocols; however, future research should consider the use of the CIEDE2000 formula to improve clinical relevance and perceptual accuracy.

Furthermore, the composition of dry mouth lozenges prescribed by dental professionals warrants scrutiny. Dentists should recommend products with non-acidic formulations that minimize harm to both dental hard tissues and restorative materials while maintaining a safe oral pH. The inclusion of calcium-based components in these products is also highly beneficial, as they support remineralization processes and contribute to the regulation of oral pH, thereby offering additional protection against demineralization and erosion [38].

## Conclusion

For pediatric patients with xerostomia requiring long-term use of saliva substitute lozenges, the choice of restorative material is critical for maintaining esthetic outcomes. This study demonstrates that composite resin restorations provide significantly superior color stability compared to compomers, which showed pronounced discoloration when exposed to certain lozenges (ACT, TheraBreath, and Biotene). The significant interaction between material type and lozenge composition indicates that clinical recommendations should consider both factors simultaneously. When compomer restorations are present, or when their use is unavoidable, lozenges with minimal discoloration potential, such as Xylimelts, should be prioritized. These findings underscore the importance of selecting both restorative materials and lozenges based on their compatibility to ensure long-term esthetic success in xerostomic patients.

## Data Availability

The entirety of the data analyzed in this study has been included in the published article. Upon making a reasonable request, the corresponding author will provide access to the raw data.
